# Peer‑led bioethics education in clinical rotations: Three generalisable lessons from a CFIR‑informed implementation case study

**DOI:** 10.1371/journal.pone.0356377

**Published:** 2026-09-29

**Authors:** Tara M. A. Shallal, Russel F. D’Souza

**Affiliations:** 1 College of Medicine, Hawler Medical University, Erbil, Kurdistan, Iraq; 2 International Chair in Bioethics, Melbourne, Australia; Kasturba Medical College Manipal, INDIA

## Abstract

**Background:**

Integrating bioethics into clinical training is challenging, particularly in resource-constrained settings. Scalable and low‑cost models are needed but are under‑evaluated. This implementation case study assessed the feasibility and perceived impact of a peer-led, case-based bioethics discussion model.

**Methodology/principal findings:**

We conducted a single embedded case study at the College of Medicine in the Kurdistan Region of Iraq. Forty-four final-year medical students participated in structured peer‑led discussions of authentic clinical ethics cases using a four‑step analytical framework. Quantitative data (post-intervention Likert questionnaire) were analyzed descriptively. Qualitative data (open‑ended responses, case summaries) underwent deductive thematic analysis guided by the Consolidated Framework for Implementation Research (CFIR) to identify barriers and facilitators across five domains. Students strongly endorsed the intervention: 59.1% strongly agreed that ethics education is important (mean 4.51/5.0). Enjoyment was high (75% agreed/strongly agreed). Self-efficacy was variable (54.5% confident, 29.5% uncertain). CFIR analysis revealed key implementation determinants: low cost and high perceived relevance (facilitators), supportive peer culture (facilitator), limited time (barrier), and need for faculty orientation (barrier). From these findings, we derived three generalizable propositions for implementing low‑cost ethics curricula.

**Conclusions/significance:**

A peer-led, case-based bioethics model is feasible and well received. Three generalizable lessons are offered: (1) peer‑led models require protected time and leveraged peer cultures; (2) single sessions raise awareness, but longitudinal reinforcement is essential for self‑efficacy; and (3) CFIR reporting enables analytical generalizability for single‑case studies. These propositions provide a blueprint for replication and further testing in low‑resource settings.

## Introduction

Bioethics education in medical curricula must move beyond theoretical frameworks to embed applied ethics in authentic clinical environments [1,2].

This shift is particularly critical in high-stakes specialties such as surgery and obstetrics-gynecology, where ethical dilemmas arise frequently and require immediate, context-sensitive responses such as effective reporting [3,4].

Constructivist and social learning frameworks emphasize active knowledge construction through real‑world engagement and collaborative dialogue [5,6]. Small group discussions and case-based learning have provided critical ethical reasoning and moral competence, as well as engaging methods of learning [7,8].

A critical dimension of clinical bioethics education involves addressing the ‘hidden curriculum’ [9]. These implicit messages can shape ethical reasoning and professional identity formation if opportunities are not structured to facilitate reflection in ways that conflict with formal educational objectives [9,10]. Therefore, students should be enabled to deliberate and examine real clinical experiences using narrative reflection as a means of processing the ethical dimensions of observed practice [5,10].

Despite the ongoing application of pedagogical approaches, a significant gap persists in scalable, student‑centred models for bioethics education during clinical rotations. This gap is especially felt in resource-constrained settings such as the Kurdistan Region of Iraq, where faculty resources and allocated curricular time are limited. Peer-led and near‑peer teaching, on the other hand, have been shown to be effective in different contexts of medical education, allowing students to drive their analysis, as an application of bioethics education, of authentic clinical cases, and could use more exploration [11,12].

We designed and piloted a structured peer‑led bioethics discussion series to address this gap and systematically evaluate it using a Consolidated Framework for Implementation Research (CFIR) [13]. This framework is used to provide a comprehensive taxonomy of constructs that influence implementation success across Innovation (the intervention itself), Outer Setting (external environment), Inner Setting (organizational context), Individuals Involved (actors’ characteristics), and Implementation Process (activities and strategies) [13,14]. Medical educationists have applied the CFIR to analyze the barriers and facilitators of curriculum adoption [15,16].

In this paper, we present a single embedded case study [17,18] of the implementation of a peer‑led, case‑based bioethics discussion model in final‑year clinical rotations at the College of Medicine in the Kurdistan Region of Iraq. Feasibility, student perceptions, and implementation determinants were evaluated using a CFIR-guided mixed-methods design to enhance analytical generalizability.

## Methods

### Study design and setting

A cross-sectional mixed-methods design was embedded as a single case study [17,18] of peer-led bioethics education that was evaluated in the College of Medicine/Hawler Medical University, Erbil, Kurdistan Region of Iraq, which follows a medical curriculum with integrated core clinical problems of case-based learning. Small-group teaching is the primary delivery method for clinical ethics, with faculty facilitating rather than directing discussions. Data were collected during the 2023–2024 academic year as part of the Surgery and Obstetrics-Gynecology clinical rotations for final-year medical students, who were recruited in the study from 1^st^ November 2023–30^th^ April 2024. No code was generated.

This study had some limitations. First, it was a single-institution study with a specific cohort and two clinical disciplines.

### Participants

Forty-four final-year medical students enrolled in their mandatory surgery rotation block provided the study population.They had all completed their core didactic bioethics curriculum and were engaged in full‑time clinical rotations. Peer-led discussion sessions were integrated into the rotation schedule and were opened with an orientation session on the importance of including ethical cases in their clinical rotation logbooks.

### The peer‑led bioethics model (innovation description)

Small groups of students (4–6 members) were organized and instructed to identify real ethical dilemmas encountered during their clinical rotations. All student facilitators were from the same final-year cohort; no senior (near-peer) students were involved. Each subgroup reviewed their clinical logbooks and selected cases representing authentic ethical challenges observed during patient care, including refusal of treatment, informed consent challenges, resource allocation dilemmas, confidentiality breaches, end-of-life decision-making, and conflicts between patient autonomy and family wishes. An example of representative vignette is: A representative case involved a patient refusing life-saving surgery on religious grounds. Students identified the ethical dilemma (autonomy vs. beneficence), explored alternative actions (respecting refusal vs. seeking psychiatric assessment), and justified their preferred course using the four-step framework.

A framework adapted from established bioethics education models [11,12] was provided to students as a standardized four‑step ethical decision‑making scheme:

(1) Problem Identification: Articulating the ethical dilemma and identifying stakeholders.
(2) Alternative Actions: Generating multiple possible courses of action and considering their ethical implications.
(3) Selection and Justification: choosing a preferred action with explicit reference to core ethical principles (autonomy, beneficence, non‑maleficence, justice); and
(4) Objections and Counterarguments: Anticipating ethical objections and formulating reasoned responses.

Each subgroup delivered a case presentation of 10 minutes followed by a 5–10 minutes of focused group discussion. Faculty facilitators (senior clinicians and medical educationists), guided case selection during preparation, provided scaffolding during discussions by posing probing questions, and offered formative feedback on presentations without imposing their own ethical conclusions.

The structured 2-hour small group intervention challenged reasoning and highlighted connections to ethical theory and professional standards.

Student performance was evaluated using a validated assessment rubric from the Undergraduate Bioethics Integrated Curriculum (Medical) 2016, developed by the UNESCO Chair in Bioethics [19]. According to this rubric, five domains were assessed: critical appraisal, group participation, humanistic attitudes, communication skills, and reflective capacity. Formative feedback was provided, and tutor and peer assessments were conducted.

### Data collection

After the intervention, a self-administered electronic questionnaire was sent to all 44 students via Google Forms employing a 5‑point Likert scale (1 = Strongly Disagree to 5 = Strongly Agree). The questionnaire included items to assess the perceived importance of bioethics education, how much they enjoyed the peer‑led format, self‑efficacy in ethical reasoning, confidence in applying ethical principles, and the prospect of expanding the experience to other departments involved in the curriculum. Additional comments and reflections were included as open‑ended questions. To document their ethical analysis based on the four‑step framework, each subgroup also generated a written case summary that was sent via email to the faculty.

### Data analysis

Mean scores, standard deviations, and frequency distributions were calculated for the quantitative data as descriptive statistics using SPSS version 25.5.

Qualitative data, which were obtained from open‑ended responses and case summaries, were analyzed in two phases. First, Braun and Clarke’s approach provided six phases of inductive thematic analysis [20] to identify emergent themes. Second, CFIR was conducted as a deductive informed analysis: the five CFIR domains (Innovation, Outer Setting, Inner Setting, Individuals Involved, Implementation Process) helped categorize the barriers and facilitators to implementation in a systematic fashion and were used as a priori coding framework [13,14]. This dual approach allowed us to derive participant insights and guide the theoretical structuring of implementation determinants.

### Ethics statement

The study was approved as part of a PhD project (Reference No. 7/1, dated July 12, 2021) by the Ethics Committee of the College of Medicine at Hawler Medical University. Implied informed consent was provided by all participants by completing and submitting the online questionnaire. This study adhered to the Strengthening the Reporting of Observational Studies in Epidemiology (STROBE) guidelines for observational studies and the Consolidated Criteria for Reporting Qualitative Research (COREQ) guidelines for qualitative research.

## Results

### Student perceptions

Forty-four students, who participated in the study completed the post-intervention questionnaire (100% response rate). Quantitative data revealed positive perceptions across multiple domains (Table 1). A strong agreement was demonstrated by the students regarding the importance of bioethics education in clinical rotations, with 59.1% strongly agreeing that ethics in surgery should be presented and discussed (mean 4.51, SD 0.63), which was the highest mean score among all items.

**Table 1:** Student perceptions of peer‑led bioethics education (N = 44).

Item	Mean (SD)	% Agree/Strongly Agree
Ethics in surgery should be presented and discussed	4.51 (0.63)	59.1% strongly agreed
I enjoyed the peer‑led session	4.02 (0.81)	75.0%
I can identify and discuss ethical issues in clinical practice	3.72 (0.95)	54.5% (29.5% uncertain)
Similar sessions should be included in other rotations	3.93 (1.02)	59.0%

*Note: Likert scale 1–5 (1 = Strongly Disagree to 5 = Strongly Agree). The percentages for the last item combined agree and strongly agree.*

With 38.6% strongly agreeing and 36.4% agreeing that they enjoyed the session (mean 4.02, SD 0.81), student enjoyment of the peer‑led format was markedly evident, while only 6.8% expressed disagreement.

There was more variability in the students’ sense of self-efficacy regarding identifying and discussing ethical issues: 54.5% agreed or strongly agreed that they could identify and discuss ethical issues in clinical practice, whereas 29.5% reported uncertainty (mean 3.72, SD 0.95). Indications of curriculum expansion were revealed through the strong agreement of 29.5% and a further 29.5% agreement (mean 3.93, SD 1.02) that Pediatrics and Obstetrics‑Gynaecology rotations could use similar sessions and should be included.

### Qualitative themes and CFIR‑informed analysis

Three principal themes were identified through thematic analysis of open‑ended responses: (1) value of practical application, (2) peer learning benefits, and (3) logistical considerations (e.g., time and resources). We mapped these themes using the CFIR domains as an a priori coding framework to identify specific constructs and barriers and facilitators. A summary of the results is presented in Table 2.

**Table 2:** CFIR domains, constructs, and implementation determinants with supporting evidence.

CFIR Domain	Construct	Barrier/Facilitator	Illustrative Quote / Evidence
Innovation	Cost	Facilitator, zero‑cost model	“No extra resources needed, just peer discussion.”
Inner Setting	Culture (peer support)	Facilitator, safe, non‑judgmental environment	“The peer‑led format created a safe space where we could share our uncertainties without fear of being judged.”
Individuals Involved	Knowledge & beliefs	Facilitator, belief in importance of ethics	Of the respondents, 59.1% strongly agreed on the importance of the program.
Implementation Process	Engaging (faculty role)	Barrier, need for faculty orientation and resources	“It would be helpful to have access to reference materials on ethical frameworks during our preparation.”
	Reflecting & evaluating	Facilitator, students provided actionable feedback	Requests for longer discussion time and better access to resources.

### Case content analysis

Three prevalent categories of ethical dilemmas were revealed from the analysis of 10 case presentations during surgery and obstetrics-gynecology rotations (Fig 1). The most frequent dilemmas (40%) were conflicts between autonomy and beneficence, involving patients who refused clinically indicated interventions, including life-saving surgery, blood transfusions on religious grounds, and discharge against medical advice. Other frequent dilemmas seen in cases (30%) revolved around informed consent and surgical scope issues, addressing challenges in emergency or time‑sensitive contexts, language barriers, and intraoperative decisions to extend procedures beyond the originally consented scope. The remaining 30% of cases, comprising justice and resource allocation dilemmas, highlighted patients inability to afford necessary diagnostic tests or procedures.

**Fig 1:**
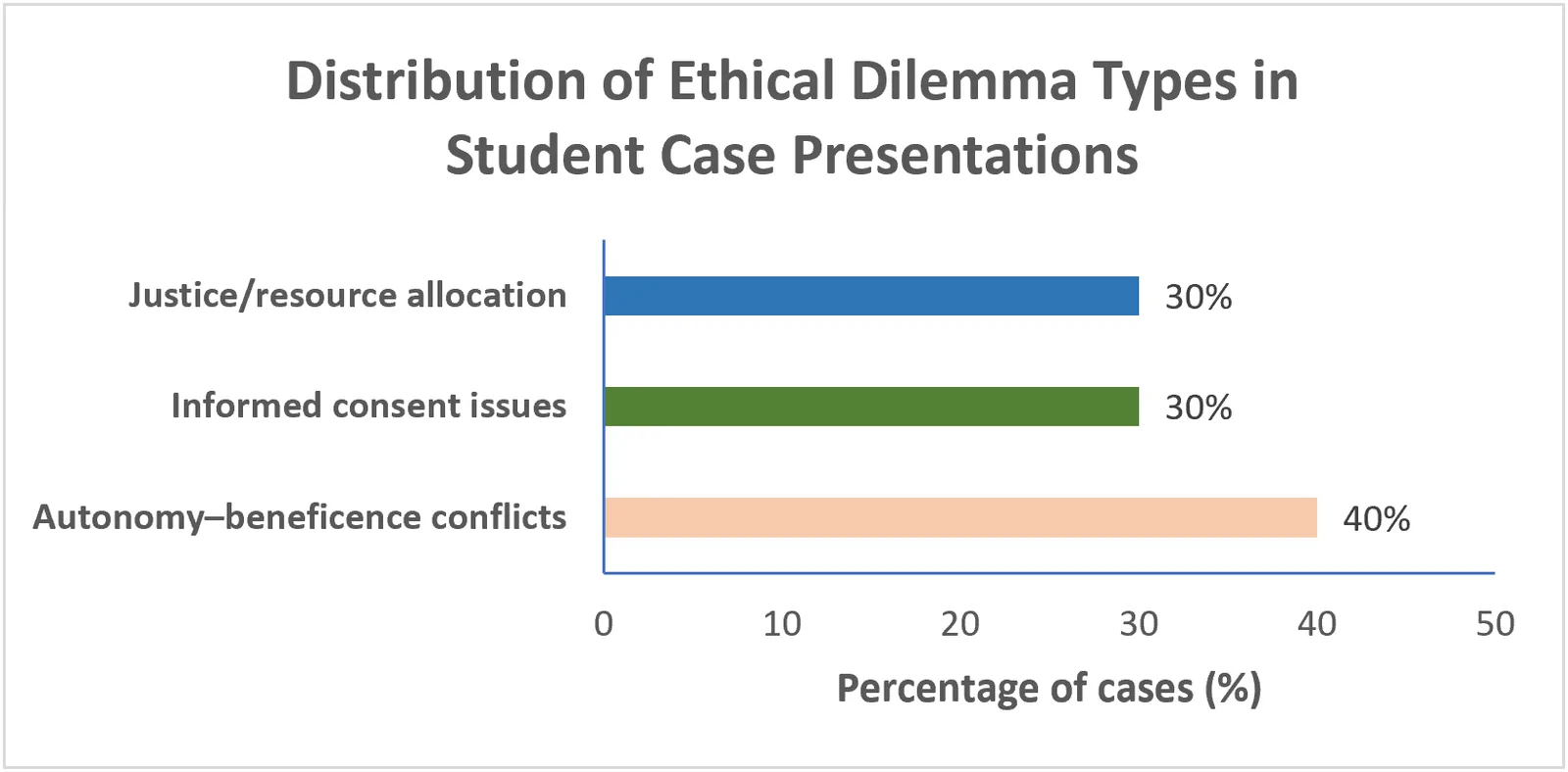
Distribution of ethical dilemma types in student case presentations (N ≈ 10 cases). Bar chart showing autonomy‑beneficence conflicts (40%), informed consent issues (30%), and justice/resource allocation dilemmas (30%). (Figure to be uploaded separately as a high‑resolution image.).

The four‑step framework offered to students demonstrated successful application in reviewing the case summaries. In addition, ethical problems should be clearly articulated, stakeholders identified, multiple alternative actions generated, core ethical principles invoked, and reasoned counterarguments to objections formulated. Students made evident through their presentations a meaningful engagement with ethical complexity rather than simplistic responses.

## Discussion

This single embedded case study used the CFIR to systematically evaluate the implementation of a peer‑led, case‑based bioethics model in a resource‑constrained setting. Below, we discuss the findings by CFIR domain and then derive three generalizable propositions for educators in similar contexts.

### Innovation domain: Why low cost and perceived value matter

The zero‑cost nature of the intervention was a major facilitator, particularly in low‑ and middle‑income countries, where educational budgets are limited. Students strongly endorsed the evidence strength and quality of the model, rating its importance as high (mean 4.51). This aligns with previous near‑peer teaching studies [21,22] and supports the analytical generalizability of the finding that low‑cost, student‑driven ethics education is both feasible and valued by students.

### Inner setting domain: Supportive culture versus structural time constraints

Peer culture, which created a psychologically safe learning environment, was a robust facilitator. Students explicitly described a safe space for discussing sensitive ethical dilemmas, a finding consistent with social learning theory. This finding aligns with Vygotsky’s Zone of Proximal Development, where peer facilitators provided scaffolding that enabled learners to achieve higher levels of ethical reasoning than they could independently [23,24]. Applying Kolb’s experiential learning cycle, students’ exposure to real ethical dilemmas provided Concrete Experience. Through peer discussion and reflection, they engaged in Reflective Observation, leading to Abstract Conceptualisation of ethical principles. This, in turn, prepares them for Active Experimentation in future clinical encounters [25].

Conversely, a major barrier was structural: insufficient allocated discussion time (less than 10 minutes per case). This reflects a common implementation challenge in packed clinical curricula [26].

### Individuals involved domain: Positive beliefs but variable self‑efficacy

Students’ strong belief in the importance of ethics education (facilitator) was tempered by variable self-efficacy (mixed). Nearly one‑third of the students remained uncertain about their ethical reasoning abilities, a finding that, rather than indicating failure, suggests that the intervention successfully raised awareness of ethical complexity without creating false confidence [27].

### Implementation process domain: Faculty orientation as a key lever

Students requested structured reference materials, a barrier that can be addressed by providing facilitator guides, case templates, ethics resource libraries, or the use of artificial intelligence.

### Three generalisable propositions from this case

Based on this single embedded case study, we propose the following propositions for testing in other contexts.

Proposition 1: Peer‑led ethics education can be implemented at zero cost, but only if existing peer relationships are leveraged as facilitators and protected time is prioritized.

Evidence: Students valued the safe space created by the peer facilitators (Inner Setting – culture). However, insufficient discussion time (less than 10 min per case) was a major barrier.

Recommendation: Allocate ≥15 minutes per case and train student facilitators to lead the discussions.

Proposition 2: A single ethics session raises awareness but does not build durable self-efficacy; therefore, ethics education must be embedded longitudinally across multiple rotations.

Evidence: 54.5% of students felt confident, but 29.5% remained uncertain (individuals’ self-efficacy). We recognise that students’ self-efficacy may vary depending on the specific ethical dilemma and their prior exposure. The uncertainty rate likely reflects the contextual nature of ethical reasoning rather than a deficit in learning. Students requested more opportunities.

Recommendation: Repeat sessions across rotations with increasing case complexity.

Proposition 3: Using CFIR to report implementation determinants enables analytical generalisability, allowing readers to compare their local context to the case and assess transferability.

Evidence: CFIR provided a structured language for barriers/facilitators.

Recommendation: Future single‑site implementation studies should adopt CFIR or similar frameworks to enhance cross‑study comparability.

### Strengths and limitations

Strengths include: the authentic clinical cases provided high ecological validity; scaffolding from structured analysis; dual peer/tutor assessment; mixed‑methods design; and explicit use of CFIR to enhance analytical generalisability.

Limitations include: single‑centre design, small sample size (N = 44), cross‑sectional data, self‑reported perceptions, absence of a control group, and lack of longitudinal follow‑up. These limit statistical generalisability but not the analytical generalisability enabled by CFIR.

### Implications for practice

CFIR‑derived barrier/facilitator Actionable recommendation.

Insufficient discussion time (Inner Setting barrier) Allocate ≥15 minutes per case; redesign session flow.

Lack of faculty orientation (Implementation Process barrier) Develop a 1‑hour facilitator guide with case examples.

Variable student self‑efficacy (Individuals mixed) Embed ethics longitudinally across multiple rotations.

Strong peer culture (Inner Setting facilitator) Leverage existing peer relationships; train student facilitators.

Zero‑cost innovation (Innovation facilitator) Emphasise this for institutional adoption.

### Future Research

Multi‑institutional replication across Iraqi medical schools using CFIR prospectively; longitudinal assessment of ethical reasoning (e.g., ethics‑focused OSCEs); evaluation at Level 4 of the Kirkpatrick framework (patient outcomes).

## Conclusion

This CFIR‑informed implementation case study demonstrates that a peer‑led, case‑based bioethics discussion model is feasible, well‑received, and perceived as educationally valuable in a resource‑constrained setting. Three generalisable propositions are offered: (1) peer‑led models require protected time and leveraged peer cultures; (2) single sessions raise awareness but longitudinal reinforcement is essential for self‑efficacy; (3) CFIR reporting enables analytical generalisability for single‑case studies. These propositions provide a blueprint for replication and further testing across low‑resource settings globally.

## Supporting information

S1 File STROBE Checklist. Completed STROBE checklist for cross-sectional studies.
(DOCX)

S2 File PLOS Inclusivity in Global Research Questionnaire. Completed questionnaire.
(DOCX)
