# Supplementary material for: Peer‑led bioethics education in clinical rotations: Three generalisable lessons from a CFIR‑informed implementation case study
Source: PLoS One. 2026 Sep 29;21(9):e0356377. doi: 10.1371/journal.pone.0356377 (PMC13623097; doi:10.1371/journal.pone.0356377)
Supplement: Supplementary file 2 [file pone.0356377.s002.docx]

**PLOS Inclusivity in Global Research Questionnaire**

Manuscript Title: Peer‑Led Bioethics Education in Clinical Rotations: Three Generalisable Lessons from a CFIR‑Informed Implementation Case Study

**Corresponding Author**: Dr. Tara M.A. Shallal

Ethical Considerations, Permits and Authorship

This section is applicable to all research types.

1. Provide details as to who granted permissions and/or consent for the study to take place in the Methods section of your manuscript. This should include the names of all ethics boards, governmental organizations, community leaders or other bodies that provided approval for the study.

Reported on page number: Methods – Ethics Statement, p. 7

Response: The study was approved by the Ethics Committee of the College of Medicine, Hawler Medical University (Reference No. 7/1, dated July 12, 2021). No other permissions were required as the study was conducted within the authors' own institution.

2. If there were any deviations from the study protocol after approval was obtained please provide details of these changes in the Methods section of your manuscript.

Reported on page number: N/A

Response: N/A – There were no deviations from the approved study protocol.

3. Did this study involve local collaborators that are residents of the country where the research was conducted or members of the community studied? If you do not have any authors from said communities, please provide an explanation for this below.

Reported on page number: Title page / Author affiliations

Response: Yes. The study was conceived, designed, and conducted entirely by local researchers at Hawler Medical University in Erbil, Kurdistan Region of Iraq. The corresponding author (Tara M.A. Shallal) is a permanent faculty member and Head of Medical Education at the institution. All participants (final‑year medical students) are residents of the Kurdistan Region of Iraq. The study was conducted within the researchers' own institution and community.

4. Everyone listed as an author should meet PLOS' criteria for authorship and all individuals who meet these criteria should be included in the author byline, rather than the acknowledgements.

Reported on page number: Title page / Author contributions

Response: Both authors meet PLOS' authorship criteria. The corresponding author (TMS) conceptualised and designed the study, collected and analysed data, and drafted the manuscript. The co‑author (RFD) provided critical revision and supervision. No eligible contributors have been placed in the acknowledgements.

Human Subjects Research

This section applies to health research, medical research, cross‑cultural psychology, and other studies involving human participants.

5. Did you obtain written informed consent from a representative of the local community or region before the research took place? How did you establish who speaks for the community?

Reported on page number: Methods – Ethics Statement, p. 7

Response: This study did not require community‑level consent as it involved an educational intervention within an academic institution. However, institutional approval was obtained from the Ethics Committee of the College of Medicine, Hawler Medical University. The study was conducted by a faculty member (the corresponding author) who is a permanent member of the institution and community, ensuring appropriate local oversight.

6. How did members of the local community provide input on the aims of the research investigation, its methodology, and its anticipated outcome(s)?

Reported on page number: N/A

Response: The research was designed by a local faculty member (TMS) with deep understanding of the local educational and cultural context. The intervention was developed in response to observed needs of final‑year medical students in the Kurdistan Region of Iraq. Student participants were directly involved in the peer‑led discussions and provided feedback through open‑ended questionnaire responses, which informed the findings and recommendations.

7. When engaging with the local community, how did you ensure that the informed consent documents and other materials could be understood by local stakeholders?

Reported on page number: Methods – Ethics Statement, p. 7

Response: Informed consent was obtained electronically via a consent statement embedded in the online questionnaire. The statement was written in plain, accessible English (the language of instruction at Hawler Medical University) and clearly explained the voluntary nature of participation, anonymity, and the purpose of the study. The questionnaire was self‑administered, and participants were free to decline or withdraw at any time.

8. Will the findings of the research be made available in an understandable format to stakeholders in the community where the study was conducted (e.g. via a presentation, summary report, copies of publications, etc.)? Please provide details of how this will be achieved.

Reported on page number: N/A

Response: Yes. The findings have already been presented at the 6th Annual Scholarship Meeting of the Global Network for Bioethics, Medical and Health Professions Education at the University of Dundee, UK (July 2026). A summary of the findings will be shared with the College of Medicine, Hawler Medical University, and made available to students and faculty. The full open‑access publication in PLOS ONE will be freely available to all stakeholders. Additionally, the corresponding author plans to present the findings to local faculty and students through institutional seminars.

Non‑Human Subjects Research

This section applies to studies using specimens/animals collected as part of the study, or those housed in archival collections. Examples include archaeology, paleontology, botany and zoology.

9. Did the permission you obtained from a local authority to perform the study include an agreement on access to outputs and benefit sharing?

Reported on page number: N/A

Response: N/A – This study did not involve non‑human subjects research, specimens, or archival collections.

10. If the material used in your study was imported, please provide the year it was imported, whether permits were obtained to import/export the materials used, and details of any permits obtained.

Reported on page number: N/A

Response: N/A – No materials were imported for this study.

11. If you used archival specimens, please state how the material used in your study was acquired by the institute it is held in and provide details of any permits obtained for the original excavations/sample collection.

Reported on page number: N/A

Response: N/A – No archival specimens were used.

12. How was the potential cultural significance of the materials collected in your study to local communities considered in your research design? Were Indigenous peoples and/or local researchers and institutions involved with archaeological excavations/collection of specimens? If so, please provide a description of their involvement.

Reported on page number: N/A

Response: N/A – This study did not involve archaeological materials, excavations, or specimen collection.

13. If your manuscript includes photographs of human remains, please indicate whether authors obtained permission from descendants or affiliated cultural communities to do so.

Reported on page number: N/A

Response: N/A – This manuscript does not include any photographs of human remains.
