# Supplementary material for: Peer‑led bioethics education in clinical rotations: Three generalisable lessons from a CFIR‑informed implementation case study
Source: PLoS One. 2026 Sep 29;21(9):e0356377. doi: 10.1371/journal.pone.0356377 (PMC13623097; doi:10.1371/journal.pone.0356377)
Supplement: Supplementary file 3 [file pone.0356377.s003.docx]

**Manuscript Number: PONE-D-26-26428**

**Full Title:** Peer-Led Bioethics Education in Clinical Rotations: Three Generalizable Lessons from a CFIR-Informed Implementation Case Study

**Abstract: Within guidelines**

**Financial Disclosure**- Nil applicable

**Competing Interests**-Nil

**Authors have competing interests**- Nil

**Ethics Statement**- Given and satisfactory

**Methodology/Principal Findings**

Please mention the pedagogic principles/andragogy principles ( as mentioned) currently followed at your centre and the nature of curriculum delivery ( socratic/PBL/case based/Competency based outcomes) .This would help colleagues from different academic contexts to familiarise with the teaching practice at the study settings.

Within the context of CIFR framework, “individuals involved” (peer led /and near peer led teaching) -were only peers from same cohort or any NPT seniors were as part of the discussion groups.

**Methods-**

**Study Design and Setting**- illustrated

Mention role of facilitators ( senior clinician and medical educationist)- During the learning process as in case selection, scaffolding during the process, assessment of summary, evaluation ?

Assessment Rubric-meets the criteria

Data Analysis- meets the criteria

Data Collection- meets the criteria

Ethics Statement- meets the criteria as per STROBE guidelines for observational studies COREQ guidelines for qualitative research.

**Results-** In my opinion , in the thematic area, students’ sense of self-efficacy reporting as uncertainty (29.5%) may be contextual and can vary from case to case basis. This could be rooted in their own belief systems and prior knowledge ( similar to those who agree ).In many ethical dilemmas, there are no absolute true or false answers. This makes the peer led discussions or clinical ethical deliberations a unique domain in ethics course delivery in medical sciences. Here the scaffolding (as in clinical apprenticeship model) by the facilitator is crucial and triangulating the observations by a third person (another facilitator) would help the learners in the process- as in given in this study.

Case content Analysis says “approximately” 10 case presentations were used - As 10 cases were only utilised , may consider removing the word “approximately”.

“*Students made evident through their presentations a meaningful engagement with ethical complexity rather than simplistic responses*.”- Ethics deliberations utilises a TYPE 2 thinking process and as a team member in the ethics discussion /deliberation team ( peer level as in here) learners leading to “meaningful engagement” during the discourse is a positive and expected outcome.

**Suggestion( Personal only**)- Application of Vygotsky’s social learning theory ( Add the Name) in andragogic settings- as in the discussion section- would also strengthen the role of peer-led and facilitated bioethics teaching in contextual learning environments as explained in this case study.

David Kolbes Theory of Experiential Learning can be adapted within the discussion section to illustrate how learners during their clinical posting went through **Concrete Experience** which led to **Abstract Conceptualisation** within the vertical axis of perception continuum; and further how their **Reflective Observations** well within the horizontal axis of processing continuum can later transform their experiences through future **Active experimentation**.

Conclusions- Are clear with feasible recommendations for further research.

PS: Thanks for this opportunity to peer review the article PONE-D-26-26428. My suggestions may be considered only if found appropriate and suitable by the authors.

Best wishes
