# Supplementary material for: Peer‑led bioethics education in clinical rotations: Three generalisable lessons from a CFIR‑informed implementation case study
Source: PLoS One. 2026 Sep 29;21(9):e0356377. doi: 10.1371/journal.pone.0356377 (PMC13623097; doi:10.1371/journal.pone.0356377)
Supplement: Supplementary file 4 [file pone.0356377.s005.docx]

Dear Academic Editor and Reviewers,

We thank the reviewers for their thoughtful and constructive comments, which have helped us strengthen the manuscript. All changes are marked with track changes.

Reviewer #1

| Comment | Response | Action |
| --- | --- | --- |
| "This is an excellent example of integrating ethical practice into case based discussions... The educational approach is well described and evaluated by a mixed methods approach." | We thank the reviewer for this positive feedback. | No action required. |
| "It would be interesting to have illustrated the report with 1 or 2 vignettes of clinical encounters discussed." | We agree. We have added a representative vignette to the Methods section. | Added a vignette: "A representative case involved a patient refusing life-saving surgery on religious grounds. Students identified the ethical dilemma (autonomy vs. beneficence), explored alternative actions, and justified their preferred course using the four-step framework." |

Reviewer #2

| Comment | Response | Action |
| --- | --- | --- |
| "Please mention the pedagogic principles/andragogy principles currently followed at your centre and the nature of curriculum delivery (socratic/PBL/case based/Competency based outcomes)." | We agree this is important context and have added a paragraph to the Methods section. | Added: "The medical curriculum at Hawler Medical University follows a competency-based model with integrated case-based learning. Small-group teaching is the primary delivery method for clinical ethics, with faculty facilitating rather than directing discussions." |
| "Within the context of CFIR framework, 'individuals involved' – were only peers from same cohort or any near-peer seniors were as part of the discussion groups?" | All facilitators were from the same cohort (peer-led). We have clarified this in the Methods. | Added: "All student facilitators were from the same final-year cohort; no senior (near-peer) students were involved." |
| "Mention role of facilitators (senior clinician and medical educationist) – during the learning process as in case selection, scaffolding during the process, assessment of summary, evaluation?" | We have expanded the description of facilitator roles. | Added: "Faculty facilitators (senior clinicians and medical educationists) guided case selection during preparation, provided scaffolding during discussions by posing probing questions, and offered formative feedback on presentations without imposing their own ethical conclusions." |
| "Case content Analysis says 'approximately' 10 case presentations were used – consider removing the word 'approximately'." | Agreed. We have removed "approximately" and now state "10 case presentations." | Changed in Results section: "Analysis of 10 case presentations..." |
| "Application of Vygotsky's social learning theory (add the name) in andragogic settings – would strengthen the role of peer-led and facilitated bioethics teaching." | We agree and have added this to the Discussion. | Added: "This finding aligns with Vygotsky's Zone of Proximal Development, where peer facilitators provided scaffolding that enabled learners to achieve higher levels of ethical reasoning than they could independently [Vygotsky, 1978]." |
| "David Kolb's Theory of Experiential Learning can be adapted within the discussion section to illustrate how learners during their clinical posting went through Concrete Experience which led to Abstract Conceptualisation..." | We have incorporated Kolb's framework as suggested. | Added: "Applying Kolb's experiential learning cycle, students' exposure to real ethical dilemmas provided Concrete Experience. Through peer discussion and reflection, they engaged in Reflective Observation, leading to Abstract Conceptualisation of ethical principles. This, in turn, prepares them for Active Experimentation in future clinical encounters [Kolb, 1984]." |
| "In the thematic area, students' sense of self-efficacy reporting as uncertainty (29.5%) may be contextual and can vary from case to case basis." | We acknowledge this important point and have added a sentence to the Discussion. | Added: "We recognise that students' self-efficacy may vary depending on the specific ethical dilemma and their prior exposure. The 29.5% uncertainty rate likely reflects the contextual nature of ethical reasoning rather than a deficit in learning." |

Journal Requirements

| Requirement | Action Taken |
| --- | --- |
| 1. PLOS ONE style requirements | Verified and formatted according to PLOS ONE templates. |
| 2. Inclusivity in Global Research Questionnaire | Completed and uploaded as Supporting Information. |
| 3. Code sharing | Statement added: "No code was generated for this study." |
| 4-5. Data Availability | Updated Data Availability Statement with ethics committee contact details. |
| 6. Supplementary figures file type | Changed file type to "Supporting Information." |
| 7. Abstract consistency | Verified abstract matches in manuscript and submission system. |
| 8. Figure 1 quality | Uploaded new high-resolution Figure 1. |
| 9. Reviewer-recommended citations | Added Vygotsky and Kolb references. |
| 10. Reference list | Verified complete and correct. |

We believe the manuscript is now significantly strengthened and hope it meets PLOS ONE's publication criteria. Thank you again for the constructive feedback.

Sincerely,

Dr. Tara M.A. Shallal
