# Supplementary material for: Peer‑led bioethics education in clinical rotations: Three generalisable lessons from a CFIR‑informed implementation case study
Source: PLoS One. 2026 Sep 29;21(9):e0356377. doi: 10.1371/journal.pone.0356377 (PMC13623097; doi:10.1371/journal.pone.0356377)
Supplement: Peer review history

# Peer review: Decision Letter 0

**DOI:** 10.1371/journal.pone.0356377.r001

**Authors:** Mary Mathew

6 Jul 2026

Dear Dr.  Shallal,

Thank you for submitting your manuscript to PLOS ONE. After careful consideration, we feel that it has merit but does not fully meet PLOS ONE’s publication criteria as it currently stands. Therefore, we invite you to submit a revised version of the manuscript that addresses the points raised during the review process.

Please submit your revised manuscript by . If you will need more time than this to complete your revisions, please reply to this message or contact the journal office at plosone@plos.org. When you're ready to submit your revision, log on to https://www.editorialmanager.com/pone/ and select the 'Submissions Needing Revision' folder to locate your manuscript file.

- A letter that responds to each point raised by the academic editor and reviewer(s). You should upload this letter as a separate file labeled 'Response to Reviewers'.
- A marked-up copy of your manuscript that highlights changes made to the original version. You should upload this as a separate file labeled 'Revised Manuscript with Track Changes'.
- An unmarked version of your revised paper without tracked changes. You should upload this as a separate file labeled 'Manuscript'.

If you would like to make changes to your financial disclosure, please include your updated statement in your cover letter. Guidelines for resubmitting your figure files are available below the reviewer comments at the end of this letter.

If applicable, we recommend that you deposit your laboratory protocols in protocols.io to enhance the reproducibility of your results. Protocols.io assigns your protocol its own identifier (DOI) so that it can be cited independently in the future. For instructions see: https://journals.plos.org/plosone/s/submission-guidelines#loc-laboratory-protocols. Additionally, PLOS ONE offers an option for publishing peer-reviewed Lab Protocol articles, which describe protocols hosted on protocols.io. Read more information on sharing protocols at https://plos.org/protocols?utm_medium=editorial-email&utm_source=authorletters&utm_campaign=protocols.

As the corresponding author, your ORCID iD is verified in the submission system and will appear in the published article. PLOS supports the use of ORCID, and we encourage all coauthors to register for an ORCID iD and use it as well. Please encourage your coauthors to verify their ORCID iD within the submission system before final acceptance, as unverified ORCID iDs will not appear in the published article. *Only*  the individual author can complete the verification step; PLOS staff *cannot*  verify ORCID iDs on behalf of authors.

We look forward to receiving your revised manuscript.

Kind regards,

Mary Mathew, MD

Academic Editor

PLOS One

Journal Requirements:

When submitting your revision, we need you to address these additional requirements.

1.Please ensure that your manuscript meets PLOS ONE's style requirements, including those for file naming. The PLOS ONE style templates can be found at

https://journals.plos.org/plosone/s/file?id=wjVg/PLOSOne_formatting_sample_main_body.pdf and

https://journals.plos.org/plosone/s/file?id=ba62/PLOSOne_formatting_sample_title_authors_affiliations.pdf

2. Please include a complete copy of PLOS’ questionnaire on inclusivity in global research in your revised manuscript. Our policy for research in this area aims to improve transparency in the reporting of research performed outside of researchers’ own country or community. The policy applies to researchers who have travelled to a different country to conduct research, research with Indigenous populations or their lands, and research on cultural artefacts. The questionnaire can also be requested at the journal’s discretion for any other submissions, even if these conditions are not met.  Please find more information on the policy and a link to download a blank copy of the questionnaire here: https://journals.plos.org/plosone/s/best-practices-in-research-reporting. Please upload a completed version of your questionnaire as Supporting Information when you resubmit your manuscript.

3. Please note that PLOS One has specific guidelines on code sharing for submissions in which author-generated code underpins the findings in the manuscript. In these cases, we expect all author-generated code to be made available without restrictions upon publication of the work. Please review our guidelines at https://journals.plos.org/plosone/s/materials-and-software-sharing#loc-sharing-code and ensure that your code is shared in a way that follows best practice and facilitates reproducibility and reuse.

4. In the online submission form, you indicated that “The data underlying the results presented in this study are available from the corresponding author upon reasonable request, subject to approval from the Research Ethics Committee of the College of Medicine, Hawler Medical University. The data are not publicly available due to ethical restrictions related to protecting participant confidentiality.”

All PLOS journals now require all data underlying the findings described in their manuscript to be freely available to other researchers, either 1. In a public repository, 2. Within the manuscript itself, or 3. Uploaded as supplementary information.

This policy applies to all data except where public deposition would breach compliance with the protocol approved by your research ethics board. If your data cannot be made publicly available for ethical or legal reasons (e.g., public availability would compromise patient privacy), please explain your reasons on resubmission and your exemption request will be escalated for approval.

5. We note that you have indicated that there are restrictions to data sharing for this study. PLOS only allows data to be available upon request if there are legal or ethical restrictions on sharing data publicly. For more information on unacceptable data access restrictions, please see http://journals.plos.org/plosone/s/data-availability#loc-unacceptable-data-access-restrictions.

Before we proceed with your manuscript, please address the following prompts:

a) If there are ethical or legal restrictions on sharing a de-identified data set, please explain them in detail (e.g., data contain potentially identifying or sensitive patient information, data are owned by a third-party organization, etc.) and who has imposed them (e.g., a Research Ethics Committee or Institutional Review Board, etc.). Please also provide contact information for a data access committee, ethics committee, or other institutional body to which data requests may be sent.

b) If there are no restrictions, please upload the minimal anonymized data set necessary to replicate your study findings to a stable, public repository and provide us with the relevant URLs, DOIs, or accession numbers. For a list of recommended repositories, please see

https://journals.plos.org/plosone/s/recommended-repositories. You also have the option of uploading the data as Supporting Information files, but we would recommend depositing data directly to a data repository if possible.

We will update your Data Availability statement on your behalf to reflect the information you provide.

6. We notice that your supplementary figures are uploaded with the file type 'Figure'. Please amend the file type to 'Supporting Information'. Please ensure that each Supporting Information file has a legend listed in the manuscript after the references list.

7. Please amend either the abstract on the online submission form (via Edit Submission) or the abstract in the manuscript so that they are identical.

8. Please upload a new copy of Figure 1 as the detail is not clear. Please follow the link for more information:  https://journals.plos.org/plosone/s/figures.

9. If the reviewer comments include a recommendation to cite specific previously published works, please review and evaluate these publications to determine whether they are relevant and should be cited. There is no requirement to cite these works unless the editor has indicated otherwise.

10. Please review your reference list to ensure that it is complete and correct. If you have cited papers that have been retracted, please include the rationale for doing so in the manuscript text, or remove these references and replace them with relevant current references. Any changes to the reference list should be mentioned in the rebuttal letter that accompanies your revised manuscript. If you need to cite a retracted article, indicate the article’s retracted status in the References list and also include a citation and full reference for the retraction notice.

[Note: HTML markup is below. Please do not edit.]

Reviewer's Responses to Questions

**Comments to the Author**

1. Is the manuscript technically sound, and do the data support the conclusions?

Reviewer #1: Yes

Reviewer #2: Yes

**********

2. Has the statistical analysis been performed appropriately and rigorously?

Reviewer #1: Yes

Reviewer #2: I Don't Know

**********

3. Have the authors made all data underlying the findings in their manuscript fully available?

The PLOS Data policy

Reviewer #1: Yes

Reviewer #2: Yes

**********

4. Is the manuscript presented in an intelligible fashion and written in standard English?

Reviewer #1: Yes

Reviewer #2: Yes

**********

Reviewer #1: This is an excellent example of integrating ethical practice into case based discussions through small group interactive learning.

The educational approach is well described and evaluated by a mixed methods approach.

It would be interesting to have illustrated the report with 1 or 2 vignettes of clinical encounters discussed.

Reviewer #2: Manuscript Number: PONE-D-26-26428

Full Title: Peer-Led Bioethics Education in Clinical Rotations: Three Generalizable Lessons from a CFIR-Informed Implementation Case Study

Abstract: Within guidelines

Financial Disclosure- Nil applicable

Competing Interests-Nil

Authors have competing interests- Nil

Ethics Statement- Given and satisfactory

Methodology/Principal Findings

Please mention the pedagogic principles/andragogy principles ( as mentioned) currently followed at your centre and the nature of curriculum delivery ( socratic/PBL/case based/Competency based outcomes) .This would help colleagues from different academic contexts to familiarise with the teaching practice at the study settings.

Within the context of CIFR framework, “individuals involved” (peer led /and near peer led teaching) -were only peers from same cohort or any NPT seniors were as part of the discussion groups.

Methods-

Study Design and Setting- illustrated

Mention role of facilitators ( senior clinician and medical educationist)- During the learning process as in case selection, scaffolding during the process, assessment of summary, evaluation ?

Assessment Rubric-meets the criteria

Data Analysis- meets the criteria

Data Collection- meets the criteria

Ethics Statement- meets the criteria as per STROBE guidelines for observational studies COREQ guidelines for qualitative research.

Results- In my opinion , in the thematic area, students’ sense of self-efficacy reporting as uncertainty (29.5%) may be contextual and can vary from case to case basis. This could be rooted in their own belief systems and prior knowledge ( similar to those who agree ).In many ethical dilemmas, there are no absolute true or false answers. This makes the peer led discussions or clinical ethical deliberations a unique domain in ethics course delivery in medical sciences. Here the scaffolding (as in clinical apprenticeship model) by the facilitator is crucial and triangulating the observations by a third person (another facilitator) would help the learners in the process- as in given in this study.

Case content Analysis says “approximately” 10 case presentations were used - As 10 cases were only utilised , may consider removing the word “approximately”.

“Students made evident through their presentations a meaningful engagement with ethical complexity rather than simplistic responses.”- Ethics deliberations utilises a TYPE 2 thinking process and as a team member in the ethics discussion /deliberation team ( peer level as in here) learners leading to “meaningful engagement” during the discourse is a positive and expected outcome.

Suggestion( Personal only)- Application of Vygotsky’s social learning theory ( Add the Name) in andragogic settings- as in the discussion section- would also strengthen the role of peer-led and facilitated bioethics teaching in contextual learning environments as explained in this case study.

David Kolbes Theory of Experiential Learning can be adapted within the discussion section to illustrate how learners during their clinical posting went through Concrete Experience which led to Abstract Conceptualisation within the vertical axis of perception continuum; and further how their Reflective Observations well within the horizontal axis of processing continuum can later transform their experiences through future Active experimentation.

Conclusions- Are clear with feasible recommendations for further research.

PS: Thanks for this opportunity to peer review the article PONE-D-26-26428. My suggestions may be considered only if found appropriate and suitable by the authors.

Best wishes

**********

what does this mean?). If published, this will include your full peer review and any attached files.

If you choose “no”, your identity will remain anonymous but your review may still be made public.

**Do you want your identity to be public for this peer review?**  For information about this choice, including consent withdrawal, please see our Privacy Policy

Reviewer #1: **Yes:** Nick Cooper

Reviewer #2: No

**********

[NOTE: If reviewer comments were submitted as an attachment file, they will be attached to this email and accessible via the submission site. Please log into your account, locate the manuscript record, and check for the action link "View Attachments". If this link does not appear, there are no attachment files.]

To ensure your figures meet our technical requirements, please review our figure guidelines: https://journals.plos.org/plosone/s/figures

You may also use PLOS’s free figure tool, NAAS, to help you prepare publication quality figures: https://journals.plos.org/plosone/s/figures#loc-tools-for-figure-preparation.

NAAS will assess whether your figures meet our technical requirements by comparing each figure against our figure specifications.

Attachment Submitted filename: PLOS PEER REVIEW.docx

# Author comment: Author response to Decision Letter 1

**DOI:** 10.1371/journal.pone.0356377.r002

2 Aug 2026

Dear Editor and Reviewers,

We thank the reviewers for their constructive comments. All reviewer-requested changes have been addressed:

We have addressed both issues requested by the journal office:

1. The Data Availability Statement now includes the email address of the Ethics Committee of the College of Medicine, Hawler Medical University (sherzad.hakim@hmu.edu.krd).

2. The reference list is correctly placed at the end of the manuscript and numbered in the order they appear in the text.

We believe these changes comply with PLOS ONE submission guidelines.

Sincerely,

Dr. Tara M.A. Shallal

Attachment Submitted filename: Response to Reviewers02.docx

# Editor report: Decision Letter 1

**DOI:** 10.1371/journal.pone.0356377.r003

**Authors:** Mary Mathew

3 Aug 2026

Peer‑Led Bioethics Education in Clinical Rotations: Three Generalisable Lessons from a CFIR‑Informed Implementation Case Study

PONE-D-26-26428R1

Dear Dr. Tara,

We’re pleased to inform you  that your manuscript has been judged scientifically suitable for publication and will be formally accepted for publication once it meets all outstanding technical requirements.

Within one week, you’ll receive an e-mail detailing the required amendments. When these have been addressed, you’ll receive a formal acceptance letter and your manuscript will be scheduled for publication.

An invoice will be generated when your article is formally accepted. Please note, if your institution has a publishing partnership with PLOS and your article meets the relevant criteria, all or part of your publication costs will be covered. Please make sure your user information is up-to-date by logging into Editorial Manager at Editorial Manager® and clicking the ‘Update My Information' link at the top of the page. For questions related to billing, please contact billing support.

If your institution or institutions have a press office, please notify them about your upcoming paper to help maximize its impact. If they’ll be preparing press materials, please inform our press team as soon as possible -- no later than 48 hours after receiving the formal acceptance. Your manuscript will remain under strict press embargo until 2 pm Eastern Time on the date of publication. For more information, please contact onepress@plos.org.

Kind regards,

Mary Mathew, MD

Academic Editor

PLOS One

Additional Editor Comments (optional):

Reviewers' comments:

# Editor report: Acceptance letter

**DOI:** 10.1371/journal.pone.0356377.r004

**Authors:** Mary Mathew

PONE-D-26-26428R1

PLOS One

Dear Dr. Shallal,

I'm pleased to inform you that your manuscript has been deemed suitable for publication in PLOS One. Congratulations! Your manuscript is now being handed over to our production team.

At this stage, our production department will prepare your paper for publication. This includes ensuring the following:

* All references, tables, and figures are properly cited

* All relevant supporting information is included in the manuscript submission,

* There are no issues that prevent the paper from being properly typeset

You will receive further instructions from the production team, including instructions on how to review your proof when it is ready. Please keep in mind that we are working through a large volume of accepted articles, so please give us a few days to review your paper and let you know the next and final steps.

Lastly, if your institution or institutions have a press office, please let them know about your upcoming paper now to help maximize its impact. If they'll be preparing press materials, please inform our press team within the next 48 hours. Your manuscript will remain under strict press embargo until 2 pm Eastern Time on the date of publication. For more information, please contact onepress@plos.org.

You will receive an invoice from PLOS for your publication fee after your manuscript has reached the completed accept phase. If you receive an email requesting payment before acceptance or for any other service, this may be a phishing scheme. Learn how to identify phishing emails and protect your accounts at https://explore.plos.org/phishing.

If we can help with anything else, please email us at customercare@plos.org.

Thank you for submitting your work to PLOS One and supporting open access.

Kind regards,

PLOS One Editorial Office Staff

on behalf of

Dr. Mary Mathew

Academic Editor

PLOS One
